# Cardiotoxicity screening of long‐term, breast cancer survivors—The CAROLE (Cardiac‐Related Oncologic Late Effects) Study

**DOI:** 10.1002/cam4.4037

**Published:** 2021-07-10

**Authors:** Lindsay L. Puckett, Shahryar G. Saba, Sonia Henry, Stacey Rosen, Elise Rooney, Samaria L. Filosa, Philip Gilbo, Karalyn Pappas, Alison Laxer, Katherine Eacobacci, Amitha N. Kapyur, Justin Robeny, Samantha Musial, Anisha Chaudhry, Rahul Chaudhry, Martin L. Lesser, Adam Riegel, Sariah Ramoutarpersaud, Navid Rahmani, Amar Shah, Vivian Papas, Toluwani Dawodu, Jessica Charlton, Jonathan P. S. Knisely, Lucille Lee

**Affiliations:** ^1^ Department of Radiation Medicine Zucker School of Medicine at Hofstra/Northwell Northwell Health Lake Success NY USA; ^2^ Department of Radiation Oncology Medical College of Wisconsin Milwaukee WI USA; ^3^ Department of Cardiology Zucker School of Medicine at Hofstra/Northwell Northwell Health Manhasset NY USA; ^4^ Department of Diagnostic Radiology Zucker School of Medicine at Hofstra/Northwell Northwell Health Manhasset NY USA; ^5^ Department of Radiation Oncology Weill Cornell Medicine New York NY USA

**Keywords:** breast cancer, cardiotoxicity, radiation, screening, survivorship

## Abstract

**Background:**

Long‐term breast cancer survivors are at risk for cardiotoxicity after treatment, but there is insufficient evidence to provide long‐term (~10 years) cardiovascular disease (CVD) screening recommendations. We sought to evaluate a tri‐modality CVD screening approach.

**Methods:**

This single‐arm, feasibility study enrolled 201 breast cancer patients treated ≥6 years prior without CVD at diagnosis. Patients were sub‐grouped: cardiotoxic (left‐sided) radiation (RT), cardiotoxic (anthracycline‐based) chemotherapy, both cardiotoxic chemotherapy and RT, and neither cardiotoxic treatment. Patients underwent electrocardiogram (EKG), transthoracic echocardiogram with strain (TTE with GLS), and coronary artery calcium computed tomography (CAC CT). The primary endpoint was preclinical or clinical CVD.

**Results:**

Median age was 50 (29–65) at diagnosis and 63 (37–77) at imaging; median interval was 11.5 years (6.7–14.5). Among sub‐groups, 44% had no cardiotoxic treatment, 31.5% had cardiotoxic RT, 16% had cardiotoxic chemotherapy, and 8.5% had both. Overall, 77.6% showed preclinical and/or clinical CVD and 51.5% showed clinical CVD. Per modality, rates of any CVD and clinical CVD were, respectively: 27.1%/10.0% on EKG, 50.0%/25.3% on TTE with GLS, and 50.8%/45.8% on CAC CT. No statistical difference was seen among the treatment subgroups (NS, χ^2^ test, *p* = 0.58/*p* = 0.15).

**Conclusion:**

This study identified a high incidence of CVD in heterogenous long‐term breast cancer survivors, most >10 years post‐treatment. Over half had clinical CVD findings warranting follow‐up and/or intervention. Each imaging test independently contributed to the detection rate. This provides early evidence that long‐term cardiac screening may be of value to a wider group of breast cancer survivors than previously recognized.

## Graphical abstract

This study identified a high incidence of cardiovascular disease in a heterogenous group of long‐term (~10 years) breast cancer survivors; over half had clinical findings warranting follow‐up and/or intervention. This provides early evidence that cardiac screening may be of value to a wider group of long‐term breast cancer survivors than previously recognized.

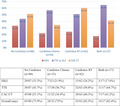

## 1 INTRODUCTION

Advancements in medicine have led to more long‐term breast cancer survivors than ever before.^1^, ^2^, ^3^ With increased longevity, long‐term or “late” adverse effects of cancer treatments are increasingly important. Common breast cancer treatments, namely radiation and chemotherapy, have known cardiotoxicities.^4^, ^5^, ^6^, ^7^ Increased cardiovascular morbidity and mortality have been detected among survivors years after diagnosis, increasing further over time.^8^, ^9^

Cardiovascular disease (CVD) screening is considered standard for some, such as pediatric cancer and lymphoma survivors.^10^, ^11^ Guidelines vary, chiefly with anthracycline and cardiac radiation dose, with higher doses linked to increased risk.^4^, ^5^, ^10^, ^11^ Prevalence of CVD on transthoracic echocardiogram (TTE) increases overtime for these populations.^4^, ^12^ Other toxicities, such as arrhythmias/conduction system disease and coronary artery disease (CAD) can be better assessed by electrocardiogram (EKG) and coronary artery calcium computed tomography (CAC CT), however, there are fewer long‐term studies that utilize these techniques. Currently in the United States (US), there is no standard ≥10‐year screening recommendation for breast cancer survivors due to the lack of prospective data to guide recommendations.^4^, ^5^ Outside the United States, a European guideline (based on expert consensus) recommends TTE screening at 10 years.^13^

Most breast cancer patients receive multiple treatments, making CVD risk assessment more challenging. A single patient may receive chemotherapy, surgery, radiation, HER‐2 targeted therapy, and up to a decade of hormonal therapy. Non‐chemotherapy, systemic treatments, such as HER‐2 targeted therapy (e.g., trastuzumab), and aromatase inhibitors can adversely impact the heart, however, are not individually considered long‐term “high/increased risk” by current guidelines.^4^, ^5^ Expert consensus remains that many breast cancer survivors could benefit from screening for CVD, but there are scant prospective data regarding timing and which modalities to employ.^4^, ^5^, ^13^

Given the range of late cardiotoxicities known to occur after breast cancer treatment, we hypothesized that a multi‐modality screening approach would be needed. Three non‐invasive, minimal risk screening studies (EKG, TTE with global longitudinal strain [TTE with GLS], and CAC CT) were chosen. We hypothesized that these tests would be feasible and appropriate for widespread screening (if ultimately indicated).

## 2 METHODS

### 2.1 Patient inclusion

This study was conducted under an IRB‐approved protocol and registered at ClinicalTrials.gov (NCT03235427). Using our institution's cancer registry, patients diagnosed with in‐situ or invasive breast cancer between 2004 and 2011 were identified. Overall, 1,144 were mailed brochures, 916 of these called for potential enrollment, 531 screened negative or were unavailable after 3 attempts, and 299 found eligible and willing, and 201 participated. Inclusion required aged 18–65 at diagnosis, ≥6 years since diagnosis, and no history of heart disease at diagnosis.

### 2.2 Enrollment

Enrollment occurred between 6/2017 and 7/2018, all patients provided written informed consent. Of the 201 enrolled, 200 had sufficient data for analysis. The enrollment aim was 200 patients on a 2:1 radiated to non‐radiated ratio over 2 years. Left‐ and right‐sided radiation patients were enrolled in roughly equal distribution (1:1). We hypothesized left‐sided, direct radiation to the heart would have a different risk than right‐sided treatment based on radiation dosimetry with standard breast/chest wall tangential field arrangements employed at our institution during the participants' treatment interval; internal mammary nodes (IMNs) were not standardly treated. We aimed to enroll 25% minority participants. Enrollment was limited to 18–65 years old at diagnosis to reduce confounding from age‐associated CVD. Recruitment began with patients with longer follow‐up (≥10 years) which created a non‐equal distribution around the mean, thus, the median is used in describing the cohort.

### 2.3 Treatment information

During screening, patients provided information regarding oncologic treatments. In almost all cases, medical records were reviewed by study staff with the patient on the phone. Treatments included: surgery, radiation, chemotherapy, hormonal therapy, HER‐targeted agents (e.g., trastuzumab), and other treatments (i.e., non‐traditional, holistic). High‐risk cardiotoxic treatments were defined as: (1) anthracycline‐based chemotherapy of greater than three cycles completed and/or known total dose of ≥300 mg/m^2^ (based on screening guidelines at the time of the study design), (2) radiation treatment of the left breast/chest wall, (3) both 1 and 2.^4^ Patients were stratified into four sub‐groups: no cardiotoxic treatment, cardiotoxic radiation, cardiotoxic chemotherapy, or both.

### 2.4 Cardiovascular risk factors

CVD was defined as prior myocardial infarction, angina, heart failure, valvular disease, wall motion abnormality, CAD, arrhythmia, pericardial disease, cardiac surgery, percutaneous coronary intervention, pacemaker or defibrillator implantation or arrhythmia ablation. Patients with cardiac risk factors without CVD at diagnosis were eligible. Potential CVD risk factors included: age at diagnosis, age at imaging, time interval from diagnosis to imaging, hypertension, smoking status, obesity, recurrence of breast cancer, chronic kidney disease, targeted therapy, hormone therapy, dyslipidemia, atrial fibrillation, history of stroke or transient ischemic attack (TIA), breast cancer treatment laterality, diabetes mellitus, family history of premature CAD (men <45 and woman <55), and ethnicity. Ethnicities were grouped as: African‐American/black, Caucasian/white, and other.

### 2.5 Cardiovascular screening

Imaging studies were evaluated with pre‐specified criteria, informed by national guidelines (Figure S1).^14^, ^15^, ^16^, ^17^ Based on the constellation of imaging findings, participants received a designation of: normal, preclinical, or clinical CVD per imaging modality. Findings that were subclinical or felt to be normal variants were tracked, but not used for clinical endpoints.

### 2.6 TTE with GLS

Echocardiograms were interpreted by two qualified board‐certified echocardiologists, COCATS Level III, Fellows of the American Society of Echocardiography, and currently hold or have held the title of Medical Director Echocardiography for a quaternary hospital system. For echocardiography, the decision to make a finding preclinical was based on minimal to mild cardiac findings that would likely not require treatment, based on cardiac expertise, but may require follow‐up with primary care physician over time. Clinical findings (based on American Society of Echocardiography Guidelines, established disease states, and cardiac expertise) were those with significant cardiac finding(s) and or at least moderate disease which likely would require follow‐up and actionable management, especially when associated with a history of breast cancer treatment. Further explanation of criteria is available in Table S1.

### 2.7 EKG

Electrocardiograms were interpreted by two well‐qualified board‐certified cardiologists. The decision to make an EKG finding preclinical was based on mild cardiac findings that would likely not require treatment, based on cardiac expertise, but may reasonably require follow‐up with a primary care physician over time. Clinical findings (based on established disease states, and cardiac expertise) were those with significant cardiac finding(s) which likely would require cardiology follow‐up and possible actionable management, especially when associated with a history of breast cancer treatment. Further details are in Table S1.

### 2.8 CAC CT

CAC CT scans were interpreted by a single board‐certified cardiologist with additional certification by the Board of Cardiovascular Computed Tomography, who held the title of Director of Cardiovascular Magnetic Resonance Imaging and Computed Tomography for a quaternary hospital system. For CAC CT, the decision to make a finding preclinical was based on a minimal to mild cardiac findings that would likely not require treatment, based on cardiac expertise, but may require follow‐up with primary care physician over time. Clinical findings (based on established disease states and cardiac expertise) were those with significant cardiac finding(s) including coronary calcium (Agatston score >0) or at least moderate disease which likely would require follow‐up and actionable management, especially when associated with a history of breast cancer treatment. Further information in Table S1.

### 2.9 Study design

The study was designed as a single‐arm, feasibility trial to assess CVD screening in long‐term breast cancer survivors. There were no prior data to inform the predicted incidence of CVD in the planned population using proposed multi‐modality imaging; formal power calculations were not planned. Study parameters were outlined with a biostatistician prior to patient enrollment. Given the expected study duration, potential participant pool, and study resources available, a 200 patient cohort was selected.

### 2.10 Primary endpoint

The presence of preclinical or clinical CVD on any imaging modality was the primary endpoint.

### 2.11 Secondary endpoints

Clinical CVD was assessed as a secondary endpoint. We also evaluated for an association between cardiotoxic radiation and/or anthracycline‐based chemotherapy and cardiac disease. Other potentially cardiotoxic treatments such as hormonal therapy and targeted agents were evaluated with univariable and multivariable analysis.

### 2.12 Statistical testing

Analysis tested for association between cardiotoxic treatments and CVD using a Chi‐Squared test. Secondary objectives included the assessment of an association between risk factors and CVD or treatment group. Associations between categorical risk factors and CVD or treatment group were analyzed using Fisher's exact test. Associations between continuous risk factors and CVD or treatment group were analyzed using the Wilcoxon Rank Sum test or the Kruskal‐Wallis test for three or more groups. Potential risk factors (see Cardiovascular Risk Factors) were analyzed in univariate analysis. Multiple logistic regression analysis was performed to test if treatment type and risk factors were significant predictors of CVD. A backward elimination method was used for the multiple logistic regression analysis to produce a model with only treatment group and significant predictors.

### 2.13 Cardiovascular risk assessment and screening

Participants underwent the assessment of weight and abdominal circumference and completed EKG (GE MAC 5500 EKG System), TTE with GLS (EPIQ Ultrasound system), and CAC CT (Canon Medical Systems, Aquilion ONE ViSION) on a single day. CAC CT studies were performed using non‐contrast, prospectively gated 320‐multidector volumetric acquisitions (DLP mean = 87.1, range = 24–159.6) with standard parameters for Agatston calcium score quantification. Total and per vessel (left main, left anterior descending, left circumflex, and right coronary artery) CAC was quantified. Strain on TTE was included as an exploratory factor as there was insufficient data to recommend for or against its use in long‐term breast cancer survivors. Evaluation was performed by three senior cardiologists (SGS, SH, SR). Patients with findings of congenital heart conditions (e.g., atrial septal defect) were marked as “unrelated.”

## 3 RESULTS

### 3.1 Patient characteristics

**FIGURE 1:**
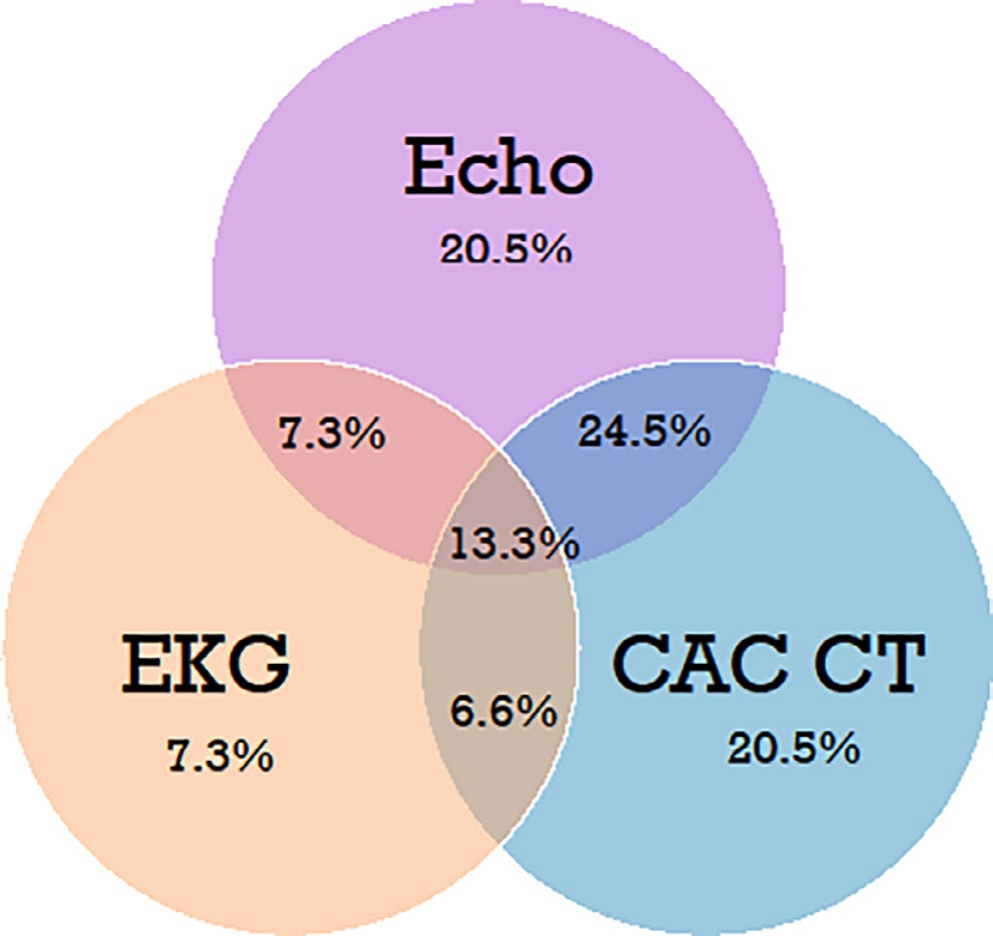
Preclinical and/or clinical disease detected per each imaging modality alone and by combined modalities among all breast cancer survivors. (%)

Median age at breast cancer diagnosis was 50 years (range 29–65) and 63 years at the time of imaging (range 37–77). Median interval from diagnosis to imaging was 11.5 years (range 6.7–14.5). Overall, 44% (*n* = 88) had no cardiotoxic treatment, 31.5% (*n* = 63) had cardiotoxic radiation only, 16% (*n* = 32) had cardiotoxic chemotherapy only, and 8.5% (*n* = 17) had both (Table 1). Among all, 77.6% had any (preclinical and/or clinical) CVD and 51.5% had clinical CVD. Per imaging modality, rates of any and clinical CVD, respectively, were: 27.1%/10.0% on EKG, 50.0%/25.3% on TTE with GLS, and 50.8%/45.8% on CAC CT. Each imaging modality contributed to the overall diagnosis and independently identified disease others did not identify (Figure 1). There was 20.5% CVD on TTE with GLS alone, 20.5% on CAC CT alone, and 7.3% by EKG alone. Many had disease on more than one modality, including 13.3% with CVD on all three tests (Figure 1). For those with CVD on TTE with GLS, GLS was the sole CVD finding for 13% (13/100).

**TABLE 1:** Participant characteristics

	No cardiotoxic treatment (*n* = 88)	Cardiotoxic chemotherapy (*n* = 32)	Cardiotoxic radiation (*n* = 63)	Cardiotoxic chemotherapy and radiation (*n* = 17)	*p*‐value
Age at diagnosis (years)	Median = 50.0 (IQR = 12.0)	Median = 48.5 (IQR = 9.5)	Median = 52.0 (IQR = 11.0)	Median = 50.0 (IQR = 10.0)	0.10
Age at imaging (years)	Median = 62.0 (IQR = 13.3)	Median = 60.7 (IQR = 8.5)	Median = 63.9 (IQR = 10.3)	Median = 59.9 (IQR = 9.8)	0.15
Time interval from diagnosis (years)	Median = 11.0 (IQR = 2.4)	Median = 12.2 (IQR = 1.8)	Median = 11.5 (IQR = 2.1)	Median = 11.9 (IQR = 2.0)	0.06
Hypertension	28/88 (31.8%)	5/32 (15.6%)	15/63 (23.8%)	5/17 (29.4%)	0.32
Smoking history	29/88 (33.0%)	7/32 (21.9%)	19/63 (30.2%)	6/17 (35.3%)	0.67
Obesity	20/87 (23.0%)	7/32 (21.9%)	17/61 (27.9%)	8/17 (47.1%)	0.22
Disease recurrence	17/88 (19.3%)	4/32 (12.5%)	18/63 (28.6%)	3/17 (17.6%)	0.30
Chronic kidney disease	1/88 (1.1%)	0/32 (0.0%)	0/63 (0.0%)	0/17 (0.0%)	1.00
Targeted therapy	6/88 (6.8%)	5/32 (15.6%)	5/63 (7.9%)	1/17 (5.9%)	0.49
Hormonal therapy	18/88 (20.4%)	12/32 (37.5%)	17/63 (27.0%)	10/17 (58.4%)	0.01
Dyslipidemia	28/59 (32.2%)	14/32 (43.8%)	22/61 (36.0%)	8/16 (50.0%)	0.43
Atrial fibrillation	3/88 (3.4%)	0/32 (0.0%)	1/63 (1.6%)	1/17 (5.9%)	0.47
TIA	3/88 (3.4%)	0/32 (0.0%)	0/63 (0.0%)	0/17 (0.0%)	0.46
Left sided breast cancer	17/87 (19.5%)	12/32 (37.5%)	63/63 (100.0%)	17/17 (100.0%)	<0.001
Diabetes mellitus	8/86 (9.3%)	4/32 (12.5%)	16/60 (26.7%)	2/17 (11.8%)	0.04
Family history of premature cardiac disease	52/87 (59.8%)	20/31 (64.5%)	39/61 (63.9%)	12/17 (70.6%)	0.86
Ethnicity (African American/Black)	5/88 (5.7%)	4/32 (12.5%)	6/63 (9.5%)	2/17 (11.8%)	0.57
Ethnicity (White/Caucasian)	75/88 (85.2%)	24/32 (75.0%)	52/63 (82.5%)	12/17 (70.6%)	
Ethnicity (all other)	8/88 (9.1%)	4/32 (12.5%)	5/63 (7.9%)	3/17 (17.7%)	

The Kruskal‐Wallis test was used to compare age and time interval by treatment group. Medians and IQRs were used for age and time interval, as these variables were not normally distributed. Fisher's exact test was used to compare categorical risk factors by treatment group. A *p*‐value of <0.05 was considered statistically significant.

### 3.2 Subgroup analysis

**FIGURE 2:**
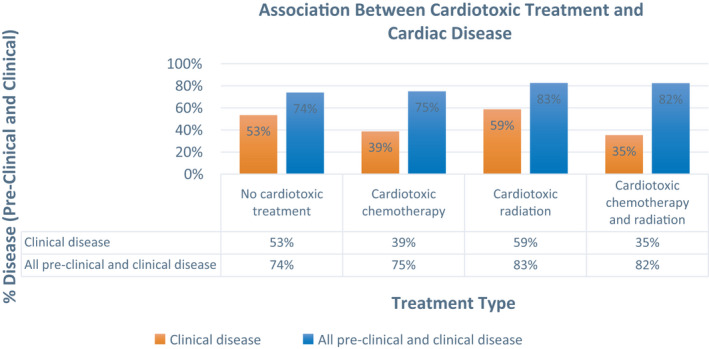
Rates of preclinical and clinical cardiac disease among treatment sub‐groups

**FIGURE 3:**
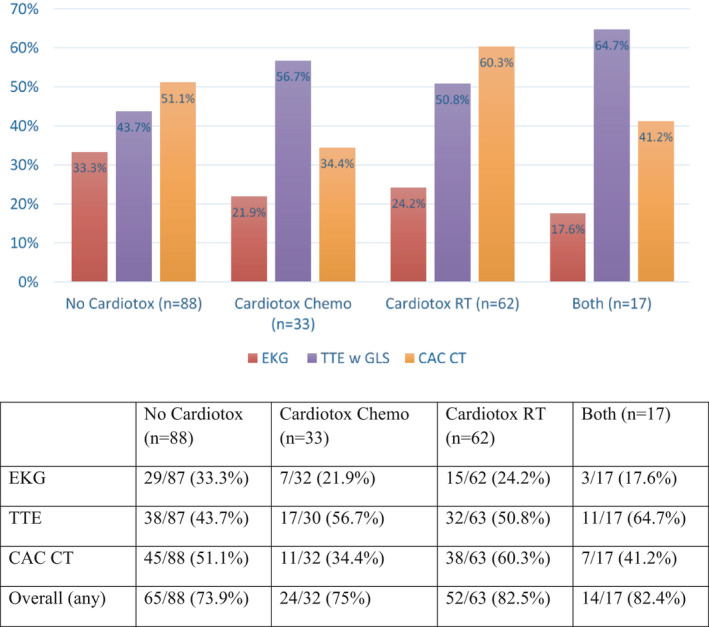
Cardiac disease incidence in treatment subgroups as found on each imaging modality. Abbreviations: CAC CT, coronary artery calcium scan; Cardiotox Chemo, cardiotoxic chemotherapy; Cardiotox RT, cardiotoxic radiation; EKG, electrocardiogram; No Cardiotox, no cardiotoxic chemotherapy or radiation; TTE w GLS, transthoracic echocardiogram with global longitudinal strain

**FIGURE 4:**
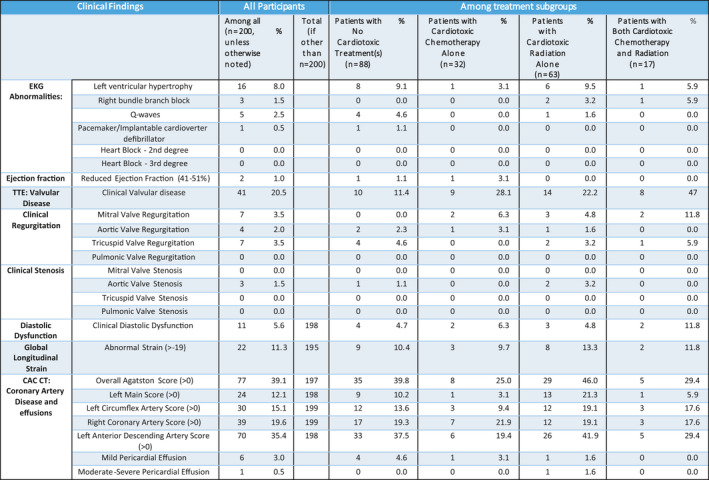
Summary of clinical findings from EKG, TTE, and CAC CT among all participants and treatment subgroups

Among subgroups, rates of any and clinical CVD detected were, respectively: 73.9%/53.4% (no cardiotoxic treatment), 82.5%/58% (cardiotoxic radiation), 75%/38.7% (cardiotoxic chemotherapy), and 82.4%/35.3% (both); rates were not statistically different between groups (χ^2^ test, *p* = 0.58/*p* = 0.15) (Figure 2). Each imaging modality contributed to disease diagnosis in each treatment subgroup (Figure 3). Additional descriptive data were collected for notable clinical cardiac toxicities such as ejection fraction, left ventricular hypertrophy (LVH), and GLS among all participants and among subgroups (Figure 4). A comprehensive list of all data including subclinical, preclinical, and clinical findings is also available (Figure S1).

### 3.3 Univariate analysis and multiple logistic regression analysis

Univariate analysis revealed that age at diagnosis (*p* < 0.0001), age at imaging (*p* < 0.0001), and hypertension (*p* = 0.022) were each associated with CVD (Table 2). All other risk factors were not significant. A multiple logistic regression model was used to analyze if treatment subgroup and risk factors were significant predictors of CVD (Table 3). A backward elimination method was used, with the treatment group as a forced variable in the model. The multiple logistic regression model revealed that age was the only significant risk factor for CVD. Controlling for other factors, increasing age was significantly associated with increased incidence of CVD (*p* < 0.0001; OR = 1.12 per year, 95% CI: 1.06–1.18), or for every 5 years, a 1.76 increased CVD risk. Cardiotoxic radiation (OR = 1.70, 95% CI: 0.57–4.87), cardiotoxic chemotherapy (OR = 1.57, 95% CI: 0.62–3.97), and both (OR = 3.14, 95% CI: 0.62–15.84) were not significant risk factors.

**TABLE 2:** Univariate analysis of post‐treatment cardiac disease by risk factors

	Preclinical or clinical cardiac disease (yes)	Preclinical or clinical cardiac disease (no)	*p*‐value
Age at diagnosis (years)	Mean = 51.60 (SD = 7.52) Median = 52.00 (IQR = 10.00)	Mean = 44.91 (SD = 7.13) Median = 44.50 (IQR = 10.50)	<0.001
Age at imaging (years)	Mean = 63.54 (SD = 7.35) Median = 63.99 (IQR = 10.73)	Mean = 56.39 (SD = 7.74) Median = 55.40 (IQR = 9.46)	<0.001
Time interval from diagnosis (years)	Mean = 11.30 (SD = 1.71) Median = 11.52 (IQR = 2.39)	Mean = 11.21 (SD = 1.87) Median = 11.51 (IQR = 2.69)	0.96
Hypertension	48/156 (30.8%)	6/45 (13.3%)	0.02
Smoking	50/156 (32.1%)	11/45 (24.4%)	0.36
Obesity	45/155 (29.0%)	7/43 (16.3%)	0.12
Recurrence	37/156 (23.7%)	5/45 (11.1%)	0.09
Chronic kidney disease	1/156 (0.6%)	0/45 (0.0%)	1.00
Targeted therapy	14/156 (9.0%)	3/45 (6.7%)	0.77
Hormone therapy	44/156 (28.2%)	13/45 (28.9%)	1.00
Dyslipidemia	61/154 (39.6%)	11/43 (25.6%)	0.11
Stroke	0/156 (0.0%)	0/45 (0.0%)	N/A
Atrial fibrillation	4/156 (2.6%)	1/45 (2.2%)	1.00
TIA	2/156 (1.3%)	1/45 (2.2%)	0.53
Breast laterality (left)	87/155 (56.1%)	22/44 (50.0%)	0.50
Diabetes mellitus	25/150 (16.7%)	5/45 (11.1%)	0.48
Family history	99/153 (64.7%)	24/44 (54.6%)	0.22
Ethnicity (AA/Black)	14/156 (9.0%)	3/45 (3.3%)	0.65
Ethnicity (White/Caucasian)	128/156 (82.1%)	36/45 (80.0%)	
Ethnicity (all other)	14/156 (9.0%)	6/45 (13.3%)	

**TABLE 3:** Multiple logistic regression model table

Multiple logistic regression model Outcome = post‐treatment cardiac disease (preclinical or clinical)						
Variable	Coefficient (β)	SE	Wald χ^2^	*p* value	Odds ratio	Odds ratio 95% CI
Intercept	−4.5572	1.3217	N/A	N/A	N/A	N/A
Cardiotoxic radiation alone	0.5125	0.5462	0.8802	0.3481	1.669	(0.572, 4.870)
Cardiotoxic chemotherapy alone	0.4545	0.4718	0.9279	0.3354	1.575	(0.625, 3.972)
Cardiotoxic chemotherapy and radiation	1.1454	0.8251	1.9269	0.1651	3.144	(0.624, 15.842)
Age at diagnosis	0.1149	0.0272	17.8654	<.0001	1.122	(1.064, 1.183)

## 4 DISCUSSION

This single‐arm, feasibility study utilized tri‐modality CVD screening to identify a high incidence of CVD in a heterogenous group of long‐term breast cancer survivors, most >10 years post‐treatment. An accrual of 200 patients was completed in 1 year at a single institution; no patient withdrew. Over half of participants had clinical CVD findings warranting follow‐up and/or intervention. Each imaging test independently contributed to the overall detection rate. All studies performed were non‐invasive and minimal risk. Those treated with high dose anthracycline or radiation treatments did not have statistically different rates of cardiac disease than these patients, with a median follow‐up of 11.5 years. These results suggest that cardiac screening may be of value to a wider group of breast cancer survivors than previously recognized.

In screening for CVD in cancer survivors, a broad range of testing modalities have been utilized.^4^, ^5^, ^10^, ^11^ The screening modalities chosen for this research (EKG, CAC CT, and TTE with GLS) were selected based on their safety and capability to detect the most commonly observed cardiotoxicities validly and reliably. Each of these studies has been used in screening studies in similar populations.^4^, ^10^, ^18^, ^19^ To our knowledge, there is no completed study that employed this combination of tri‐modality screening.

Electrocardiograms are widely accessible, cost‐effective, and a potential screening method for asymptomatic patients at risk for CVD.^19^ In a large prospective trial of asymptomatic, post‐menopausal women (*n* = 14,749, mean age = 63), 28% of patients had minor EKG abnormalities and 6% had major abnormalities; clinically relevant EKG abnormalities were independently associated with an increased risk of cardiovascular events and mortality.^19^ Within the CAROLE study (median age = 63) with both pre‐ and post‐menopausal patients, we found overall a 27.1% incidence of preclinical and/or clinical disease and 10.0% clinical disease on EKG. EKG independently and uniquely identified CVD in 7.3% of participants that had otherwise not been classified as having CVD. Interestingly, among those without cardiotoxic chemotherapy or left‐sided radiation, 33% of participants had abnormalities on EKG (Figure 3), the most of any subgroup. This finding warrants further exploration to assess whether there is a specific subset (e.g., right‐sided radiation and/or non‐anthracycline chemotherapy(s) patients) especially at risk who contributed to that finding. Given the low‐cost and simplicity of interpretation, others have also concluded that EKG is a useful screening tool in predicting future cardiovascular events in asymptomatic post‐menopausal women.^19^

CAC CT has also been shown to serve as a valid and reliable test for detecting CVD.^20^, ^21^ The availability of CAC CT has increased significantly in recent years and is appropriate to cancer survivors at risk for CAD.^20^, ^21^, ^22^, ^23^ Those who received radiation to the heart are at higher risk for CAD, but are not standardly screened.^24^ In our cohort, CAC CT independently identified an equal amount of disease to that identified with the more standard screening modality of TTE (20.5% each modality) (Figure 1), suggesting its utility in long‐term breast cancer survivors. In one study of newly diagnosed, high cardiac risk breast cancer patients, 26% (mean age = 60) had positive CAC.^24^ In our cohort (median age = 63), 38.6% of participants had positive CAC alone, and 45.8% had evidence of any CVD (including non‐CAC) on CAC CT. The rate was >60% in those with left‐sided radiation (Figure 3). Allowing for differences in age and risk factors, these rates are substantially higher than historical controls and concerning in a population not generally screened for coronary artery disease.^25^

The most common screening method for evaluating cancer therapy‐related cardiac dysfunction is TTE.^4^, ^5^, ^10^, ^11^, ^26^, ^27^ This imaging technique is widely used in detecting abnormal wall motion and allows providers to mitigate CVD risk by serving as a potential intervention tool, particularly in anthracycline‐treated patients.^26^, ^28^, ^29^ It has been shown to be a cost‐effective, accessible, valid, and reliable measure of cardiac disease.^26^, ^27^ In our study cohort, 50% of participants had preclinical or clinical CVD on TTE with GLS; interestingly, rates were also high among groups that did not receive high‐dose anthracycline treatments (no cardiotoxic treatment = 43.7%, cardiotoxic radiation = 50.8%, Figure 3).

Recently, GLS has been evaluated for use in cancer patients.^28^, ^29^ In our study, 13.5% of participants had abnormal GLS, ranging from 11% to 15% among treatment groups (Figure S1); further analysis with a larger cohort is indicated. Notably, GLS testing identified 13% of TTE‐delineated CVD (13/100) which otherwise may have been missed.

Over the past decade, the European Society for Medical Oncology (ESMO) (2012) and the American Society of Clinical Oncology (ASCO) (2017) released guidelines to help direct post‐treatment cardiac screening for adult cancer survivors.^4^, ^5^ Among these, the two most well‐established risks for late‐effect CVD are left‐sided radiation and anthracycline‐based chemotherapy. We found patients who did not receive either of those high‐risk treatments still displayed high rates of CVD (73.9% preclinical and clinical, and 53.4% clinical alone). This group included those with right‐sided radiation, low (<300 mg) and non‐anthracycline‐based chemotherapies, trastuzumab use, hormonal treatments, and surgery alone.

There is a scant study of breast cancer patients who did not receive cardiotoxic agents within oncology literature. We postulated that this group would serve as an internal control within our study as they were not exposed to these treatment‐related CVD risk factors. Surprisingly, we observed high rates of CVD among this group, beyond that previously seen in untreated, age‐matched peers.^19^, ^24^ Thus, we postulate that a larger group of survivors than previously anticipated may have increased risk of CVD.

It may well be that simply being a breast cancer survivor increases cardiac risk. Recent data from a SEER analysis of 300,000 cancer survivors (>65 years), including ~35% breast cancer survivors, showed that they were more likely to develop new cardiovascular morbidity compared to controls with no prior cancer diagnosis.^30^ This was consistent with an analysis of >36,000 patients in a large managed care organization and others' reports.^31^, ^32^ There are numerous overlapping risk factors between heart disease and breast cancer (e.g., age, obesity, diet, hormone replacement).^12^ Indirect cardiac effects of cancer treatment(s) may include decreased long‐term fitness, exercise intolerance, and an associated increased cardiac risk.^33^ While screening risks also exist, this must be weighed against the harms of undiagnosed and potentially treatable CVD. In this cohort of 200 breast cancer survivors, without known CVD at diagnosis, over half had findings that warranted further work‐up and/or intervention.

This study has several limitations. To our knowledge, the CAROLE Study is the first to employ the described tri‐modality screening approach, thus baseline rates of expected CVD were not established nor were formal power calculations conducted. Our assessments of cardiac disease were uniform, pre‐determined, and utilized standard practices whenever possible (Table S1), however, practice patterns vary nationally and internationally. Inter‐observer variability in the delineation of CVD is likely. Cardiologists were blinded to the treatment sub‐group; however, aware they were oncologic patients and study participants which may bias interpretation.

Participants were treated from 2004 to 2011; since then, radiation techniques have significantly reduced cardiac dose (i.e., DIBH, prone, and field‐in‐field). After 2009, lower mean heart doses are expected; however, this represents a small subset of our cohort.

While our results suggest the value of screening breast cancer survivors, this finding should be further confirmed with a group of matched non‐oncology patients. Prospective studies are currently underway but long‐term data are not expected for ~10 years. Based on our data and other recent literature, we hypothesize that most women treated for breast cancer ‐including those without cardiotoxic chemotherapy or radiation‐ are at increased risk of CVD. We postulate that further study is needed to ensure long‐term survivors are appropriately screened for CVD risks and disease.^5^, ^30^

## CONFLICT OF INTEREST

None.

## ETHICS STATEMENT

This study was approved by the Northwell Health IRB.

## Supporting information

Fig S1

Table S1

## Data Availability

The data that support the findings of this study are available from the corresponding author upon reasonable request.
