# Supplementary material for: Cardiotoxicity screening of long‐term, breast cancer survivors—The CAROLE (Cardiac‐Related Oncologic Late Effects) Study
Source: Cancer Med. 2021 Jul 10;10(15):5051–61. doi: 10.1002/cam4.4037 (PMC8335805; doi:10.1002/cam4.4037)
Supplement: Supplementary file 1 — Fig S1 [file CAM4-10-5051-s002.pdf]

Clinical Findings		All Participants			Among treatment subgroups							
		**Among all (n=200, unless otherwise noted)**	**%**	**Total (n)**	**Patients with No Cardiotoxic Treatment(s) (n=88)**	**%**	**Patients with Cardiotoxic Chemotherapy Alone** (n=32)	**%**	**Patients with Cardiotoxic Radiation Alone (n=63)**	**%**	**Patients w/ Both Cardiotoxic Chemo and Radiation (n=17)**	**%**
EKG findings: Rhythm	*Normal Sinus Rhythm*	*184*	*92.0*	*200*	*82*	*93.2*	*30*	*93.8*	*56*	*88.9*	*16*	*94.1*
	Sinus Bradycardia	9	4.5	200	4	*4.6*	1	3.1	4	6.4	0	0.0
	Sinus Tachycardia	0	0.0	200	0	*0.0*	0	0.0	0	0.0	0	0.0
	Atrial Fibrillation	0	0.0	200	0	*0.0*	0	0.0	0	0.0	0	0.0
	Atrial Flutter	0	0.0	200	0	*0.0*	0	0.0	0	0.0	0	0.0
	Supraventricular tachycardia	0	0.0	200	0	*0.0*	0	0.0	0	0.0	0	0.0
	Premature ventricular contractions	1	0.5	200	0	*0.0*	0	0.0	0	0.0	1	5.9
Other EKG Findings	Heart Block - 1st degree	5	2.5	200	1	*1.1*	1	3.1	3	4.8	0	0.0
	Heart Block - 2nd degree	0	0.0	200	0	*0.0*	0	0.0	0	0.0	0	0.0
	Heart Block - 3rd degree	0	0.0	200	0	*0.0*	0	0.0	0	0.0	0	0.0
	Right bundle branch block	3	1.5	200	0	*0.0*	0	0.0	2	3.2	1	5.9
	Left bundle branch block	2	1.0	200	0	*0.0*	2	6.3	0	0.0	0	0.0
	Abnormal QT interval	5	2.5	200	4	*4.6*	0	0.0	0	0.0	1	5.9
	Abnormal QTc interval	6	3.0	200	4	*4.6*	0	0.0	1	1.6	1	5.9
	Left ventricular hypertrophy	16	8.0	200	8	*9.1*	1	3.1	6	9.5	1	5.9
	Right ventricular hypertrophy	0	0.0	200	0	*0.0*	0	0.0	0	0.0	0	0.0
	Left atrial abnormality	8	4.0	200	6	*6.8*	0	0.0	2	3.2	0	0.0
	Right atrial abnormality	2	1.0	200	1	*1.1*	0	0.0	1	1.6	0	0.0
	ST elevation	0	0.0	200	0	*0.0*	0	0.0	0	0.0	0	0.0
	ST depression	3	1.5	200	3	*3.4*	0	0.0	0	0.0	0	0.0
	Non-specific T-wave Changes	15	7.5	200	8	*9.1*	1	3.1	5	7.9	1	5.9
	T wave inversion	3	1.5	200	3	*3.4*	0	0.0	0	0.0	0	0.0
	Q-waves	5	2.5	200	4	*4.6*	0	0.0	1	1.6	0	0.0
	Low Voltage	4	2.0	200	2	*2.3*	0	0.0	2	3.2	0	0.0
	Pacemaker/Implantable cardioverter defibrillator	1	0.5	200	1	*1.1*	0	0.0	0	0.0	0	0.0
	Other EKG	7	3.5	200	5	*5.7*	1	3.1	1	1.6	0	0.0
EKG Impressions	EKG Impression- Preclinical disease	34	17.2	198	18	*20.7*	5	15.6	9	14.5	2	11.8
	EKG Impression- Clinical disease	20	10.1	198	11	*12.6*	2	6.3	6	9.7	1	5.9
Echocardiogram findings: Pericardial Disease	*Subclinical Pericardial Disease*	*9*	*4.5*	*200*	*6*	*6.8*	*1*	*3.1*	*1*	*1.6*	*1*	*5.9*
	Clinical Pericardial Disease	6	3.0	200	4	*4.6*	2	6.3	0	0.0	0	0.0

	Pericardial Calcification	0	0.0	200	0	0.0	0	0.0	0	0.0	0	0.0
	Pericardial Constriction	0	0.0	200	0	0.0	0	0.0	0	0.0	0	0.0
	Pericardial Effusion	6	3.0	200	4	4.6	2	6.3	0	0.0	0	0.0
	Cardiac Tamponade	0	0.0	200	0	0.0	0	0.0	0	0.0	0	0.0
Hypokinesis	Anterior Hypokinesis	0	0.0	200	0	0.0	0	0.0	0	0.0	0	0.0
	Septal Hypokinesis	4	2.0	200	2	2.3	1	3.1	1	1.6	0	0.0
	Lateral Hypokinesis	0	0.0	200	0	0.0	0	0.0	0	0.0	0	0.0
	Posterior/Inferior Hypokinesis	4	2.0	200	2	2.3	1	3.1	1	1.6	0	0.0
	Apical Hypokinesis	0	0.0	200	0	0.0	0	0.0	0	0.0	0	0.0
Ejection Fraction	Normal Ejection Fraction (<52%)	2	1.0	200	1	1.1	1	3.1	0	0.0	0	0.0
	Mildly Reduced Ejection Fraction (41-51%)	2	1.0	200	1	1.1	1	3.1	0	0.0	0	0.0
Cardiomyopathy	Cardiomyopathy (any)	9	4.6	196	4	4.6	2	6.3	1	1.6	2	11.8
Global Valvular Calcification and Thickening	*Subclinical (MAC, >70 yrs)*	*25*	*12.5*	*200*	*16*	*18.2*	*1*	*3.1*	*5*	*7.9*	*3*	*17.6*
	Clinical Mitral Annular Calcification	19	9.5	200	3	3.4	3	9.4	7	11.1	6	35.3
	Mitral Valve Calcification	4	2.0	200	0	0.0	1	3.1	1	1.6	2	11.8
	Aortic Valve Calcification	18	9.0	200	5	5.7	4	12.5	3	4.8	6	35.3
	Tricuspid Valve Calcification	1	0.5	200	0	0.0	1	3.1	0	0.0	0	0.0
	Pulmonic Valve Calcification	0	0.0	200	0	0.0	0	0.0	0	0.0	0	0.0
	Mitral Valve Thickening	*7*	*3.5*	*200*	*2*	*2.3*	*2*	*6.3*	*3*	*4.8*	*0*	*0.0*
	Aortic Valve Thickening	*4*	*2.0*	*200*	*2*	*2.3*	*0*	*0.0*	*2*	*3.2*	*0*	*0.0*
	Tricuspid Valve Thickening	*0*	*0.0*	*200*	*0*	*0.0*	*0*	*0.0*	*0*	*0.0*	*0*	*0.0*
	Pulmonic Valve Thickening	*0*	*0.0*	*200*	*0*	*0.0*	*0*	*0.0*	*0*	*0.0*	*0*	*0.0*
Stenosis	*Trivial Stenosis of the Mitral Valve*	*0*	*0.0*	*200*	*0*	*0.0*	*0*	*0.0*	*0*	*0.0*	*0*	*0.0*
	Trivial Stenosis of the Aortic Valve	*1*	*0.5*	*200*	*0*	*0.0*	*0*	*0.0*	*1*	*1.6*	*0*	*0.0*
	Trivial Stenosis of the Tricuspid Valve	*0*	*0.0*	*200*	*0*	*0.0*	*0*	*0.0*	*0*	*0.0*	*0*	*0.0*
	Trivial Stenosis of the Pulmonic Valve	*0*	*0.0*	*200*	*0*	*0.0*	*0*	*0.0*	*0*	*0.0*	*0*	*0.0*
	Trivial Stenosis- Not specified	*1*	*0.5*	*200*	*1*	*1.1*	*0*	*0.0*	*0*	*0.0*	*0*	*0.0*
	Mild Stenosis of the Mitral Valve	0	0.0	200	0	0.0	0	0.0	0	0.0	0	0.0
	Mild Stenosis of the Aortic Valve	2	1.0	200	0	0.0	0	0.0	2	3.2	0	0.0
	Mild Stenosis of the Tricuspid Valve	0	0.0	200	0	0.0	0	0.0	0	0.0	0	0.0
	Mild Stenosis of the Pulmonic Valve	0	0.0	200	0	0.0	0	0.0	0	0.0	0	0.0
	Moderate Stenosis of Heart Valves	0	0.0	200	0	0.0	0	0.0	0	0.0	0	0.0
	Severe Stenosis of the Mitral Valve	0	0.0	200	0	0.0	0	0.0	0	0.0	0	0.0

	Severe Stenosis of the Aortic Valve	1	0.5	200	1	1.1	0	0.0	0	0.0	0	0.0
	Severe Stenosis of the Tricuspid Valve	0	0.0	200	0	0.0	0	0.0	0	0.0	0	0.0
	Severe Stenosis of the Pulmonic Valve	0	0.0	200	0	0.0	0	0.0	0	0.0	0	0.0
	Other Mitral Valve Stenosis	0	0.0	200	0	0.0	0	0.0	0	0.0	0	0.0
	Other Aortic Valve Stenosis	1	0.5	200	1	1.1	0	0.0	0	0.0	0	0.0
	Other Tricuspid Valve Stenosis	0	0.0	200	0	0.0	0	0.0	0	0.0	0	0.0
	Other Pulmonic Valve Stenosis	0	0.0	200	0	0.0	0	0.0	0	0.0	0	0.0
**Regurgitation**	Trivial Regurgitation	39	19.5	200	16	18.2	9	28.1	11	17.5	3	17.6
	Trivial Aortic Regurgitation	5	2.5	200	1	1.1	1	3.1	3	4.8	0	0.0
	Trivial Tricuspid Regurgitation	18	9.0	200	9	10.2	5	15.6	2	3.2	2	11.8
	Trivial Pulmonic Regurgitation	2	1.0	200	0	0.0	1	3.1	1	1.6	0	0.0
	Unspecified Valve- Trivial Regurgitation	29	14.5	200	12	13.6	4	12.5	8	12.7	5	29.4
	Mild Mitral Valve Regurgitation	24	12.0	200	11	12.5	3	9.4	9	14.3	1	5.9
	Mild Aortic Valve Regurgitation	7	3.5	200	1	1.1	2	6.3	3	4.8	1	5.9
	Mild Tricuspid Valve Regurgitation	11	5.5	200	3	3.4	3	9.4	3	4.8	2	11.8
	Mild Pulmonic Valve Regurgitation	1	0.5	200	1	1.1	0	0.0	0	0.0	0	0.0
	Moderate Mitral Valve Regurgitation	7	3.5	200	0	0.0	2	6.3	3	4.8	2	11.8
	Moderate Aortic Valve Regurgitation	4	2.0	200	2	2.3	1	3.1	1	1.6	0	0.0
	Moderate Tricuspid Valve Regurgitation	7	3.5	200	4	4.6	0	0.0	2	3.2	1	5.9
	Moderate Pulmonic Valve Regurgitation	0	0.0	200	0	0.0	0	0.0	0	0.0	0	0.0
	Severe Regurgitation	0	0.0	200	0	0.0	0	0.0	0	0.0	0	0.0
	Other Regurgitation	0	0.0	200	0	0.0	0	0.0	0	0.0	0	0.0
**Diastolic Dysfunction**	Subclinical Diastolic Dysfunction (E/A reversal and >60 yrs old)	30	15.2	198	15	17.1	4	12.5	10	15.9	1	5.9
	Clinical Diastolic Dysfunction	11	5.6	198	4	4.6	2	6.3	3	4.8	2	11.8
**Global Longitudinal Strain**	Abnormal Strain (>-19)	22	11.3	195	9	10.4	3	9.7	8	13.3	2	11.8
**Pulmonary Hypertension**	Subclinical Pulmonary Hypertension (35-39 mm Hg)	6	5.0	119	3	5.8	0	0.0	2	5.3	1	9.1
	Clinical Pulmonary Hypertension	4	3.4	119	2	3.9	0	0.0	2	5.3	0	0.0
**Echocardiogram Overall Impression**	Echo Impression- Preclinical Disease	48	24.4	197	19	21.8	9	28.1	14	22.2	6	35.3
	Echo Impression- Clinical Disease	50	25.4	197	19	21.8	8	25.0	18	28.6	5	29.4
**CAC CT findings**	Mild Mitral Annular Calcification	32	16	199	12	13.6	2	6.3	15	23.8	3	17.6
	Moderate-Severe Mitral Annular Calcification	2	1.0	199	1	1.1	1	3.1	0	0.0	0	0.0
	Aortic valve calcification	39	19.6	199	14	15.9	3	9.4	17	27.0	5	29.4

	Overall Agatston Score (>0)	77	39.1	197	35	39.8	8	25.0	29	46.0	5	29.4
	Left Main Score (>0)	24	12.1	198	9	10.2	1	3.1	13	21.3	1	5.9
	Left Circumflex Artery Score (>0)	30	15.1	199	12	13.6	3	9.4	12	19.1	3	17.6
	Right Coronary Artery Score (>0)	39	19.6	199	17	19.3	7	21.9	12	19.1	3	17.6
	Left Anterior Descending Artery Score (>0)	70	35.4	198	33	37.5	6	19.4	26	41.9	5	29.4
	Mild Pericardial Effusion	6	3.0	200	4	*4.6*	1	3.1	1	1.6	0	0.0
	Moderate-Severe Pericardial Effusion	1	0.5	200	0	*0.0*	0	0.0	1	1.6	0	0.0
CAC CT Overall Impression	CAC Impression – Preclinical Disease	10	5.0	200	4	*4.6*	1	3.1	3	4.8	2	11.8
	CAC impression- Clinical Disease	91	45.5	200	41	*46.6*	10	31.3	35	55.6	5	29.4

Subclinical or within range of normal – tracked for research, but not sufficient for endpoints of preclinical or clinical disease

Data collected but not intended for the study's cardiac endpoints (not shown above): Pericardial fat pad, Thickened pericardium, Isolated basal inferior hypokinesis (normal variant), MAC Agatston score, AV Agatston score, Aortic root Agatston score, Aortic valve calcification, Descending thoracic aorta Agatston score, Caliber Aorta in oblique plane, Caliber Aorta in axial plane, Caliber Main Pulmonary Artery, Lipomatous hypertrophy, Lipomatous infiltration of the interatrial septum, Intramyocardial fat, Epicardial fat thickness, Qualitative size of heart chambers, Lung abnormality, Hiatal hernia, Esophageal abnormality, Lymph node abnormality, Liver abnormality, and Other abnormalities)

Supplemental Figure 1. Detailed report of tracked cardiac imaging findings from EKG, TTE, and CAC CT and overall impression per modality.
