# Supplementary material for: Cardiotoxicity screening of long‐term, breast cancer survivors—The CAROLE (Cardiac‐Related Oncologic Late Effects) Study
Source: Cancer Med. 2021 Jul 10;10(15):5051–61. doi: 10.1002/cam4.4037 (PMC8335805; doi:10.1002/cam4.4037)
Supplement: Supplementary file 2 — Table S1 [file CAM4-10-5051-s001.docx]

**Supplemental Table 1. Description of normal, preclinical, and clinical disease per imaging modality**

**Echocardiogram (TTE)**

| **TTE Findings** | **Normal TTE** | **Preclinical TTE** | **Clinical TTE** |
| --- | --- | --- | --- |
| Ejection Fraction ^1^ | Normal: EF > 52% |  | EF<52% |
| Pericardial disease ^2^ | Normal pericardium | Pericardial fat pad  Thickened pericardium | Pericardial calcification  Pericardial constriction  Pericardial effusion  Pericardial tamponade |
| Coronary artery disease (suspected ischemic cardiomyopathy/segmental wall motion) | Normal Wall motion | isolated basal inferior or basal inferoseptal hypokinesis (normal variant) | Any segments wall motion abnormalities, except isolated basal inferior or basal inferoseptal hypokinesis |
| Global Cardiomyopathy | None |  | Any global cardiomyopathy i.e. Restrictive myopathy, dilated Cardiomyopathy, RV dysfunction, other Cardiomyopathy. |
| Valvular disease (Description/Structure)  I.e. thickening, calcification, caseous mitral annular calcification | Normal | Mitral annular calcification age >70^3^  Thickening of leaflets (isolated)  Annular calcification (isolated)  Mitral valve prolapse | Moderate calcification (leaflet and/ or annulus)  Calcification on leaflet and annulus on one valve <age 60 |
| Valvular stenosis^4^ | None | Trivial disease | Mild, Moderate, severe stenosis of any valve(a) |
| Valvular regurgitation^5^ | Trivial regurgitation  Mild regurgitation (isolated)  Mild Tricuspid (isolated) |  | > Moderate regurgitation of any valve(s) |
| Diastolic dysfunction^6^ | Normal | E/A reversal age>60 | Mild <age 60, Moderate, Severe diastolic dysfunction |
| Global longitudinal strain (GLS) on Philips Epic ^7^ | Normal GLS <-19 |  | GLS >-19 |
| Pulmonary Hypertension^8^ | Normal <35mmmHg | 34-49mmHg | >50 mmHg, Moderate, Severe pulmonary hypertension |
| Other findings |  | Hyper mobile septum | Dilated left atrium |

1. <https://www.asecho.org/wp-content/uploads/2016/02/2015_ChamberQuantificationREV.pdf> (J Am Soc Echocardiogr 2015;28:1-39.
2. https://www.asecho.org/wp-content/uploads/2014/02/2013_Multimodality-CV-Imaging-for-Pericardial-Disease.pdf (J Am Soc Echocardiogr 2013;26:965-1012.)
3. [Heart.](https://www.ncbi.nlm.nih.gov/pmc/articles/PMC1767558/) 2003 Feb; 89(2): 161–164. PMID: [12527666](https://www.ncbi.nlm.nih.gov/pubmed/12527666)
4. <https://www.asecho.org/wp-content/uploads/2014/05/2009_Echo-Assessment-of-Valve-Stenosis.pdf> PMID: 28363204
5. <https://www.asecho.org/wpcontent/uploads/2017/04/2017VavularRegurgitationGuideline.pdf> PMID: 28314623
6. <https://www.asecho.org/wp-content/uploads/2016/03/2016_LVDiastolicFunction.pdf> J Am Soc Echocardiogr. 2016 Apr;29(4):277-314. PMID: 27037982
7. Normal GLS for Philips using Qlab was – 18.9. Lang RM,et al JASE 2015; 28:1-39
8. <https://www.asecho.org/wp-content/uploads/2013/05/Echo-Assessment-of-Right-Heart-in-Adults.pdf> PMID: 20620859

**Electrocardiogram (EKG)**

| **Normal EKG** | **Preclinical EKG** | **Clinical EKG** |
| --- | --- | --- |
| Normal Sinus Rhythm  Sinus Bradycardia  Sinus Tachycardia | Premature ventricular contractions  Premature atrial contractions  Heart Block - 1st degree  Right bundle branch block  Non-specific T-wave Changes  T wave inversion | Atrial Fibrillation  Atrial Flutter  Supraventricular tachycardia  Heart Block - 2nd degree  Heart Block - 3rd degree  Right bundle branch block  Left bundle branch block  Abnormal QT interval  Abnormal QTc interval  Left ventricular hypertrophy  Right ventricular hypertrophy  Left atrial abnormality  Right atrial abnormality  ST elevation  ST depression  Q-waves  Low Voltage  Pacemaker/defibrillator |

**Coronary Artery Calcium Computed Tomography Scan (CAC CT)**

| **Normal CAC CT** | **Preclinical CAC CT** | **Clinical CAC CT** |
| --- | --- | --- |
| Total Agatston Score = 0  No Aortic Valve Calcification  No Mitral Annular Calcification  No Calcification of Thoracic Aorta  Normal Caliber Ascending Aorta  No pericardial effusion | Minimal or Mild Aortic Valve Calcification  Minimal or Mild Mitral Annular Calcification  Minimal or Mild Calcification of Thoracic Aorta | Total Agatston Score >0  Left Main Calcium Subtotal >0  Left Anterior Descending Calcium Subtotal >0  Left Circumflex Calcium Subtotal >0  Right Coronary Artery Calcium Subtotal >0  Moderate or Severe Aortic Valve Calcification  Moderate or Severe Mitral Annular Calcification  Moderate or Severe Calcification of Thoracic Aorta  Dilated Ascending Aorta  Pericardial Effusion |
